# Guideline Standardisation, Cost Effectiveness, Industry Needs and Conflict of Interest

**DOI:** 10.4103/0973-1229.32150

**Published:** 2007

This section reports the findings and recommendations of an important Conference on Guidelines Standardisation (COGS). It also looks into why there is a discrepancy between, for example, CPG and CDR recommendations while discussing the recent case of a new long acting insulin (*insulin gargline)*. It also considers cost-benefit calculations for CPGs, should or should not CPGs perform economic analysis, currently funded therapy and cost effectiveness, budget information and economic assessment, universal effectiveness and variable cost effectiveness etc. It then looks into how CPGs are made to service Industry interests and what are the various issues of conflict of interest in CPGs.

## Conference On Guideline Standardisation (COGS): An Important Initiative

It may help to look into an important initiative on guideline standardisation here. This is one serious effort to support the process of improving guideline quality, as well as help point out the ones that lack it. Rather than pick on faults and chastise, it would create a situation wherein faults become unacceptable and evidence and quality necessarily the norm. Hence this initiative needs more than a cursory look.

A Conference on Guideline Standardisation (COGS) was convened in April 2002 ‘*to define a standard for guideline reporting that would promote guideline quality and facilitate implementation*’ (Shiffman *et al.*, 2003).

Twenty-three people with expertise and experience in guideline development, dissemination and implementation participated. Representatives of 22 organisations active in guideline development reviewed the proposed items and commented favourably.

Closely related items were consolidated into 18 topics to create what they called the COGS Checklist. (Shiffman *et al.*, 2003). These items are:

1. ‘Overview material’, which provides ‘a structured abstract that includes the guidelines release date, status (original, revised, updated) and print or electronic sources’.
2. ‘Focus’, which describes ‘the primary disease/condition and intervention/service/technology that the guideline addresses, indicate any alternative preventive, diagnostic or therapeutic interventions that were considered during development’.
3. ‘Goal’, which describes ‘the goal that following the guideline is expected to achieve, including a rationale for development of a guideline on this topic.’
4. ‘User/setting’, which describes ‘the intended users of the guidelines (e.g., provider types, patients) and the settings in which the guideline(is) intended to be used.’
5. ‘Target population’, which describes ‘the patient population eligible for guideline recommendations and lists any exclusion criteria’.
6. ‘Developer’, which identifies ‘the organization(s) responsible for guideline development and the names/credentials/potential conflicts of interest of iindividuals involved in the guideline's development’.
7. ‘Funding source/sponsor’, which identifies ‘the funding source/sponsor and describe(s) its role in developing and/or reporting the guideline. Disclose conflict of interest.’
8. ‘Evidence collection’, which describes ‘the methods used to search the scientific literature, including the range of dates and databases searched and criteria used to filter the retrieved evidence.’
9. ‘Recommendation grading criteria’, which describes ‘the criteria used to rate the quality of evidence that supports the recommendations and the system for describing the strength of the recommendations. Recommendation strength communicates the importance of adherence to a recommendation and is based on both the quality of the evidence and the magnitude of anticipated benefits or harms’.
10. ‘Method for synthesizing evidence’, which describes ‘how evidence was used to create recommendations e.g. evidence tables, meta-analysis, decision analysis.’
11. ‘Prerelease review’, which describes ‘how the guideline developer reviewed and/or tested the guidelines prior to release.’
12. ‘Update plan’, which states ‘whether or not there is a plan to update the guideline and if applicable, an expiration date for this version of the guideline’.
13. ‘Definitions,’ which ‘define unfamiliar terms and those critical to correct application of the guideline that might be subject to misinterpretation’.
14. ‘Recommendations and rationale’, that ‘state the recommended action precisely and the specific circumstances under which to perform it. Justify each recommendation by describing the linkage between the recommendation and its supporting evidence. Indicate the quality of evidence and the recommendation strength, based on the criteria described in 9’.
15. ‘Potential benefits and harm’, which ‘describe anticipated benefits and potential risks associated with implementation of guideline recommendations.’
16. ‘Patient preferences’, which describes ‘the role of patient preferences when a recommendation involves a substantial element of personal choice or values’.
17. ‘Algorithm’, which provides ‘(when appropriate) a graphic description of the stages and decisions in clinical care described by the guideline.’
18. *‘* Implementation considerations’, which ‘describe anticipated barriers to application of the recommendations. Provide reference to any auxiliary documents for providers or patients that are intended to facilitate implementation. Suggest review criteria for measuring changes in care when the guideline is implemented’(Shiffman *et al.*, 2003).

A careful look at the items reveals the comprehensive nature of this Checklist. Each item needs more than cursory perusal by those interested in salvaging CPGs from ulterior influence. Please do not just skim through them. Read and discuss how each item is important, so they register and maybe you may add a few to them yourself.

It includes items for standardization (Items 5,6,8,9,10), conceptual issues (Items 2,3,13,14), up gradation (Items 1,12,18), conflict of interest (Items 6,7), patient interest (Items 4,5,11,15,16,18) and systematization (Items 4,8,9,10,13,14,17,18). Even items for individual preferences, choice or values are not neglected (Item 16). Special mention must be made of Items 6 and 7, which specify disclosure of conflict of interest both in the Developer (including the organization that develops and the individuals involved in the guideline's formulation), as well as in the sponsor or funding source (and its role in developing and/or reporting the guideline). All salutary moves.

Another significant contribution of this checklist is its prospective use:

In contrast to other instruments that have been developed for post hoc evaluation of guideline quality, the COGS checklist is intended to be used prospectively by developers to improve their product by improving documentation (Shiffman et al., 2003).

Also, it is the result of a consensus standard for prospective development of quality CPGs:

Although many individual organizations have devised manuals and procedures for developing guidelines, we are unaware of any consensus standard that has been proposed for use prospectively to promote the development of high-quality guidelines (Shiffman et al., 2003).

The paper rightly ends with a call to overcome roadblocks in CPG use by carrying on a ‘sustained’ and ‘productive’ discussion amongst all the parties involved: developers, disseminators, implementers and ‘knowledge managers’ of guidelines:

A sustained and productive discussion among guideline developers, disseminators, implementers and knowledge managers about critical guideline items and clear statement of decidable and executable recommendations will help to overcome major impediments to guideline use (Shiffman et al., 2003).

COGC is an important initiative in the right direction. Those interested in salvaging CPGs from market forces camouflaging as evidence-based medicine may neglect it at their own peril.

## Discrepancy Between CPG And CDR, Recommendations: Why?

However, useful as the COGC initiative is, there is no mention of any reasons for discrepancy between, for example, CPG and Common Drug Review (CDR) recommendations. Nor any cost-benefit calculations. In forwarding a sustained and productive discussion, some attention may profitably be devoted to these two hitherto neglected areas.

This comes to attention as one studies the conflicting recommendations of CPGs and Drug review panels. This is the case in Canada, but similar problems must arise in other places too. An interesting rather recent such disparity was in 2003 and reported in an Editorial of the *CMAJ* (*CMAJ*, 2005). It is worth a small elaboration here.

An expert committee of the Canadian Diabetes Association (CDA) responsible for laying down CPGs recommended that, for treatment of Type I and Type II Diabetes patients where there were problems of fasting hypoglycemia and/or problems controlling fasting plasma glucose, a new long acting insulin, insulin gargline, be used in preference to generic long-acting insulin in use till date (Canadian Diabetes Association Clinical Practice Guidelines Expert Committee, 2003). In 2005, members of the Common Drug Review (CDR), a Canadian national advisory process that evaluates drugs for provincial formularies, recommended that the drug not be listed (CMAJ, 2005; Canadian Coordinating Office for Health Technology Assessment, 2005). ‘Both groups of experts evaluated virtually the same evidence from about 20 randomized controlled trials’ (CMAJ, 2005).

The Association sponsored CPG recommends a drug. The Drug Review Panel rejects the same drug. Both come to their conclusions from the same set of clinical trials. How is that possible?

The CDA guidelines did not disclose whether members of their expert panel had financial or other interests linked with manufacturers of the preferred insulin gargline. But the CDA was quick to refute CDR's recommendations against insulin gargline. They sent an open letter to provincial ministries of health (addressed to the health minister) expressing concern with the CDR recommendation (Canadian Diabetes Association, 2007). Also worth noting here is the fact that insulin gargline is three times costlier than generic long-acting insulin. (The above quoted editorial mentions five times, but they corrected themselves later.)

That same editorial quotes a recent report on more than 200 guidelines (from various countries) deposited in 2004 with the US National Guideline Clearinghouse, which found that “more than one third of the authors declared financial links to relevant drug companies, with around 70% of panels being affected.” (Taylor and Giles, 2005). It goes on to make a piquant comment:

This controversy over guidelines is not unique to those developed by the CDA. There has been similar debate regarding the management of hypertension, where national guidelines recommend expensive patented drugs over proven generic compounds. Almost all consensus and guideline development panels are supported by pharmaceutical companies with vested interests and many panelists receive research grant support and personal compensation for lectures and advice from the same companies… To maintain that such financial conflicts exert no influence on panelists' recommendations is to ignore accumulating evidence that they do. A national guideline recommendation encumbered by money in the form of lecture and consulting fees, stocks, options, patents and royalties may be effective in increasing sales and profits for companies, but may also be harmful to patients. And it will almost always result in higher-cost drugs being prescribed. (According to the CDR, insulin glargine costs 5 times as much as generic long-acting insulin*.) (CMAJ, 2005)

Before we jump to the conclusion that this is another clear case of manipulation by pharma, let us also look into an argument presented cogently by one who has been on both panels, a CPG and a Drug Review Panel. Phil McFarlane, from the Division of Nephrology, St. Michael's Hospital, Toronto, Ontario, Canada, is ‘one of the few physicians who have served on both clinical practice guideline groups and drug review panels (in his case the CDA and Canadian Hypertension Education Program [CHEP] guideline groups and the Ontario Drug Programs Branch Pharmacoeconomic Review Committee respectively) (McFarlane, 2006). This is what he has to say:

The mandate of guideline groups and drug review panels differ so extensively that one should expect that their respective conclusions will often differ. Guideline groups advocate use of the most effective therapies as suggested by the medical literature and typically do not perform economic analyses when generating guidelines. Drug review panels determine whether a new therapeutic is sufficiently cost-effective and has an acceptable budget impact within the context of their jurisdiction (McFarlane, 2006).

He then goes on to list four primary reasons why guidelines do not and in his opinion should not, carry out economic analyses:

First, guideline groups do not have a mandate from any provincial or federal agency to make decisions about what therapies will be publicly funded. Equally important, they have no mandate to recommend removal of currently funded therapeutics when the cost-effectiveness of care would benefit from such an action. Second, guideline groups are not provided projected budget information that would help inform an economic assessment. Third, one could consider an assessment of effectiveness to be somewhat “universal.” In contrast, the determination of whether a therapy is acceptably cost-effective can certainly vary between jurisdictions. Finally, an economics based approach would place guideline groups in a true conflict of interest between their patient advocacy role and their obligations to the health care payors (McFarlane, 2006).

The points he makes are so important that they deserve a detailed analysis. Let us take them up, one by one.

## Cost-Benefit Calculations For CPGs

Let us take up the first point about the basic difference between Guideline Groups and Drug Review Panels here:

The mandate of guideline groups and drug review panels differ so extensively that one should expect that their respective conclusions will often differ. Guideline groups advocate use of the most effective therapies as suggested by the medical literature and typically do not perform economic analyses when generating guidelines. Drug review panels determine whether a new therapeutic is sufficiently cost-effective and has an acceptable budget impact within the context of their jurisdiction (McFarlane, 2006).

In essence, this means Guideline groups look into the most effective therapies, Drug review panels look into the most *cost* effective therapies. Which means we realize and accept, that the most therapeutically effective may not necessarily also be the most cost effective. And the most cost effective may not necessarily be the most therapeutically effective. In the context of effectivity, cost should not act as a constraint, especially when as important an issue as therapy and potential morbidity and mortality are involved. Therefore, it makes sense to allow Guidelines groups to do their job. However, it is equally important cost effectiveness be looked into, since spending is an issue, especially for statutory and federal authorities. So, it makes sense for them to carry out cost calculations and determine most cost effective therapies, which they do with their Drug Review Panels. So, the prescribing doctor writes the CPG recommended most effective treatment when cost is not a constraint. And writes the CDR recommended one when it is.

So far so good. And this can be a beautiful resolution of the issue for all concerned. Except that there is a spanner in the works.

## Spanner In The Works

In determining that which is the most therapeutically effective, what considerations enter into the picture? Obviously, the results of therapeutic trials and the discretion of giving weightage proportionate to the methodological rigour of the trials considered. But what do we do when most CPGs, as reported earlier, do not adhere to established methodological standards? When critical information that would attest to validity is regularly absent from CPGs? When explicit criteria to grade the scientific evidence that supports their recommendations is absent from 82% of guidelines? When 87% are not in a position to report whether a systematic literature search was performed? When 67% do not describe the type of professionals used in guidelines development? When there is such marked variation in the quality of guidelines? And both ‘nonadherence to methodologic standards’ and ‘failure to document development activities’ contribute to this variation?

And how unequivocal are their findings?

To find an answer to this, consider the following. What happens when the results of these trials are manipulated, the methodological rigour reported is more on paper than in practice and the unequivocal findings are based on fudged figures at industry centers rather than in academic setups? Since many of the latter are only too happy to hand over findings to industry personnel to do as suits them, as long as publication in a prestigious journal is arranged? What happens when the CPG panelists themselves face a major conflict of interest, being in financial compromise with the very companies whose products are being considered? What do we make of the fact that most CPGs recommend products that belong to the companies sponsoring the CPG panelists?

Here is the problem. And a big one at that.

But this problem can be conveniently resolved by a simple step. By making CPG panelists go into cost effectiveness along with recommending Guidelines. What then happens is that they have to consider not only effectiveness but also costs. Now effectiveness can be fudged, cost cannot. Why? Because, what is the cost is well known. When Guidelines are recommended and they are graded according to whether they are Most, Moderately or Least Cost Effective, some very salutary processes are set into motion.

Panelists cannot neglect the cheaper alternatives under the pretext of effectivity. Panelists cannot tout the most recent as the most effective, as often they are markedly costly compared to the earlier. And they will have to say this in so many terms. In fact that is the reason newer Guidelines are a flourishing academic activity. Panelists will then have to get themselves funded for their fringe, and other, benefits by the most cost effective, if at all. And that is hardly likely to work, for they will not then remain cost effective. (There is of course the danger that the most cost effective will attract such self-seekers. But in trying to pamper the latter, they may no longer remain cost effective. So they will be forced not to cater to the panelists’ interests to protect their own).

Moreover, the costlier processes are then likely to be exposed for what they are. If some people still desire them, as well they might (for in some cases the more costly the procedure the greater its appeal), let it remain the preserve of such self-gratification seekers. For the rest who count their rupees (or dollars, whatever) and have to pay from their hard earned and scarce resources, it makes sense for CPG panelists to lay down not only effective but cost effective guidelines. And those who count their rupees of course include insurance companies and government bodies, but equally important, paying patients.

## Resistance To Be Expected

We are aware this will be resisted, for so many interests are at stake. It just takes the wind out of their sales. If cost effectiveness enters the calculations of payers like insurance companies and government bodies, as well as individual patients, the most recent costly medication can hardly come on the most preferred list. And become the potential money-spinner for the company marketing it. The move will be resisted tooth and nail by all sorts of ostensibly plausible arguments pitted against it. They will try their level best to camouflage their private concerns as common welfare. And evidence based medicine. But it is time those who have the larger interests of effective biomedical advance and genuine patient welfare understand the stakes involved, the game being played and seek to nullify its ulteriority (without of course blunting its legitimate thrust).

This is one cat and mouse game well worth playing, even if basic reluctance and disgust at the goings on prompt skepticism and dismay and often the tendency to give up the fight.

## Should Or Should Not CPGs Perform Economic Analysis?

Let us now come to the four primary reasons why, according to the above author, Guideline Groups should not perform economic analysis:

There are 4 primary reasons why guideline groups do not (and in my opinion should not) perform economic analyses when generating guidelines. First, guideline groups do not have a mandate from any provincial or federal agency to make decisions about what therapies will be publicly funded (McFarlane, 2006).

This situation can be countered by simply giving them such a mandate. What if they had such a mandate? Would they carry out such a cost benefit analysis? This is a question which needs a serious answer. Our hunch is they would say no for a variety of reasons meant to camouflage the most important one which will never be mentioned: the commercial interest of sponsors. One sometimes feels why don't the guys make a clean breast of it and end this whole debate? Just say: yes. We have, and need, sponsors. We cannot neglect their commercial interests. We must include the prominent sponsor's product in our guidelines. So what's your problem? If you have objections, go raise them at whatever forum and suit yourself. End of debate.

Well and truly, debate would just end. But something salutary would start. The lid would be off the whole can of worms.

Then the real fight could begin. The large number of ethically conscious observers, whose major energies are today directed towards exposing such malevolence, could wait a while and contemplate on the fundamental issue as to where should medicine head. Whether it should become a corporate enterprise, remain a profession or become a professional enterprise. Something we deliberated on in an earlier monograph (Singh and Singh, 2005-2006).

## Why Restrict Cost-Benefit Analysis To Publicly Funded Therapies?

But to continue with the analysis at hand. Why should a cost-benefit analysis be restricted only to publicly funded therapies? Simply because federal agencies do it anyway. If others start doing it as well, it's checkmate for sponsors and their benefactors. Every guideline follower would have data of cost effectiveness with every guideline. Sponsors would have to keep costs of products down. How is that possible with such huge overheads and huge profits to be made by each new product? The game would be over even before it was played.

Just think of it another way. If you, or we, had to pay for a certain therapy, would we not want to have an informed opinion on which is the most cost effective? Should the fact that they have/don't have such a mandate from any provincial or federal agency be the only reason why a cost-benefit analysis need be carried out? Let us not kid ourselves. Somebody is paying for somebody using the recommended guideline. Why can that individual/agency not have the most cost-effective remedy spelt out?

The game of justifying why no economic analysis is to be carried out when generating guidelines is not easy to decipher. Simply because if they did carry it out, the cat would be instantly out of the bag. For the cost dynamics of the new treatment regimen would be immediately exposed. Trust the Guideline layers to resist any such move. If the cost of the new therapy were substantially lower than the earlier or equivalent and found more effective, they would have no compunctions agreeing to such a move. But, it is a hidden assumption that every new therapy will be substantially costlier than the earlier and hence needs scientifically appropriate justification, without highlighting the significant cost escalation involved, for that would lead to disastrous end user resistance.

## Intrinsic Therapeutic Worth May Be Genuinely Costly

Let us consider another argument. What about recommending a therapy for its intrinsic therapeutic worth? That it is costly is unfortunate, but incidental. That is not in control of guideline formulators. That is for authorities and companies who market it to decide. And on marketing departments which gauge how much the market will profitably sustain. An expert exercise in itself. But outside the domain of expertise of guideline formulators.

This would be laudable if therapies were recommended solely because of their intrinsic therapeutic worth. That would happen only if guidelines developers were not conflicted in their interests and considered the benefits of a certain recommendation without favour and only after a careful appraisal of research evidence of the clear cut effectiveness of a certain recommended therapy as against one which is not. What evidence is given weightage is very important here. When that itself is liable to manipulation, when newer therapies are projected better not in comparison to older therapies but as compared to placeboes, what other method of control remains except to consider cost effectiveness? It is a tragedy that the only evidence that has remained objective is not scientific evidence but the cost factor. You should not be able to, but unfortunately can, manipulate scientific evidence. You cannot manipulate declared cost, which is for all to see. Well, smart operators will manipulate that as well, when they calculate how cost is not just money, but also time, smoothness of the procedure, distress etc. But that can be seen through by the rights-conscious market savvy patient of today. So, we really have no option today but to include cost dynamics in guideline recommendations.

We need not mind even if CPGs could be rated on a sliding economic scale. There are some for whom nothing less than the costliest will do. Well, they have a choice, as do their caretakers. Why not make it all explicit, so those who seek cost effectiveness get it and others who seek cost exclusivity get that. Like someone wants to stay in a five star or seven star, willingly goes in for the cost of the same. Why should a person who cannot afford five star therapy be forced into it, simply because a guideline cannot offer him a cheaper alternative? The patient, and his treating physician/hospital, is forced to think the CPG offered is the best and he must bear its exorbitant cost. If he is offered an alternative wherever possible, well, his caretakers and he himself, will have the option to decide which, for him. Then, if he/they decide to go in for the costliest, well, it's their outlook. It will be a truly informed choice.

## Currently Funded Therapy And Cost Effectiveness

Equally important, they have no mandate to recommendremoval of currently funded therapeutics when the cost-effectiveness of care would benefit from such an action (McFarlane, 2006).

What does that mean? Have they ever demanded the mandate *to 'recommend removal of currently funded therapeutics when the cost-effectiveness of care would benefit from such an action’?* Now or ever? What prevents them from making such a demand explicit? Why this is important is because when cost effectiveness becomes an important feature to remove current therapy, it becomes equally important, by default, an important reason to approve new therapy. And therein lies the rub. It is important, therefore, that those involved in laying down new/updated CPGs be given the mandate to 'recommend removal of currently funded therapeutics when the cost effectiveness of care would benefit from such an action’ as much as given the mandate to recommend inclusion of a new therapy *only* when the cost effectiveness would benefit from such an action.

Why? Because, such cost calculation automatically helps to keep a check on cost escalation and all the questionable activities it can help spawn. It also helps CPG panelists keep to the straight course. And it also helps treating physicians and their patients benefit from such cost calculation.

It is imperative that treating physicians and paying patients know the costs that therapy involves. And if a newer one is low on the cost effectiveness scale, the physician is well aware of the same and recommends treatment accordingly. As it happens, physicians may not ordinarily make such calculations while recommending treatment, implicitly assuming that the more costly, the better. A reflection of the same is the attitude of the patient/care giver who reassures his physician not to bother about costs, but carry out treatment, however costly it may be. Now, we know, some patients/caregivers are only reassured if the costliest therapy is given, even if they may not afford it. That is to quell any subsequent guilt feelings that the ‘best’ was not given simply because it was costly. But if the costly is not really the best, is it not the duty of the treating physician to make it explicit? And how, pray, will he ever make it explicit if he himself is not aware of the costs and benefits involved, which experts can, and should, guide him about? So, while he is open to new therapies, the hype over new treatments does not carry him away?

If he keeps to the straight and narrow path, and if his CMEs allow him to, what really would happen is, he would prescribe the best possible treatment at the most cost effective rates. But to do so, he himself should have carried it out. And for him to carry it out, the smart alecks who carry out his CMEs should carry it out and present it as such. For them to do so, the smarter alecks who lay down CPGs must carry it out. However, if they do, they are hardly likely to forward the profit welfare agenda of their sponsors. So, the convenient method is not to involve CPGs in any cost benefit analysis at all. Just leave it to the discretion of individual practitioners who will conveniently err on the cost escalation side to appear more informed. Or expect the governmental and other statutory bodies to carry out such cost effective analysis. But that is hardly likely to influence the great mass of prescribers. Firstly, because they may hardly know about it, since the visibility/credibility of such analysis is low compared to the marketing and ‘evidential’ pressures of the smart alecks. Secondly, because to go by the cost effective guidelines may hardly be the in, or fashionable, thing to do. So the smart alecks manage to set and dictate the agenda, and do so on ostensibly justified scientific grounds: that CPGs do not, and should not, have the mandate or the necessity to carry out cost benefit analysis.

Well, if you want a better and subtler method of exploitation and all so justifiably camouflaged, you are hardly likely to find one.

## Budget Information And Economic Assessment

Let us take up the second point:

Second, guideline groups are not provided projected budget information that would help inform an economic assessment (McFarlane, 2006).

Indeed they are not. But do they ask for budget information? Now let us ask this question. If Guidelines were not provided with evidence based research studies, would they work? Obviously not, because they consider that integral to their work. If they were to consider cost benefit analysis as important to their work as research evidence, they would refuse to work in the absence of such budget information. Admitted, they may not be experts in such an analysis. They need not be. They could have on their committee someone who is. In fact, an expert who carries out cost benefit analysis of every Guideline recommendation should be an integral part of the committee. He brings in the economic dimension, while the other experts bring in the medical. It is of course best if the medical expert is also an expert economic analyzer, but that is expecting a little too much in every case. In the circumstances, such an expert will carry out his economic analysis. The whole committee can then deliberate over all issues - fresh evidence available, costs involved and intended beneficiaries of such CPGs.

They may then do well to lay down the following:

1. Most cost effective;
2. Moderately cost effective;
3. Least cost effective.

Let there be some exasperation over this process. Let there also be some heated discussion over it. But let it be done, so caregivers/patients/ practitioners can decide the best options for those to be cared for.

## A Whole Paradigm Shift

The moment those who have to lay down guidelines have to think thus, there is a whole paradigm shift in thinking involved. CPG panelists will have to necessarily carry out cost calculations as well, something far from their minds at present. The new treatments, to get recommended, will have to keep their costs down. The artificial jacking up, the proliferation of 'me-toos’ cannot remain justified, for they will get discussed and exposed at the Guidelines level itself. And manufacturers/pharmaceuticals will have to justify costs or face boycott. Moreover, the industry-expert nexus cannot proliferate as it does today.

What happens at present is very convenient. The manufacturer funds experts for their activities. The experts sit on committees and recommend their products. The consumer is not offered any cost analysis but impressive portrayals in seminars and journal articles. So the costly new process gets acceptance. And milks patients and payers till a new process can be found to milk them further.

Now, it does not mean there is no benefit with the present procedure. If that were so, the game would have been exposed by now already. And we would have no need to write pages over it. It is just that the benefits are not commensurate with the costs involved. And, to keep the game under wraps, perceptions are adroitly changed. What appeared the most promising till yesterday is suddenly found full of faults today. It falls out of favour, for a new star comes on the ascendant. We have detailed this game before (Singh and Singh, 2003). And prescribers and beneficiaries (patients) get taken for a ride. We do not suggest cost benefit analysis is the panacea to this problem. But it will be one important step forward to stem the rot.

However, let's not forget that it will meet with great, and apparently justified, resistance. For the forces that will not get a chance to play their game know exactly what this means. They will present arguments how it is not in the benefit of biomedical research or patient welfare for experts to carry out cost analysis. That's for caretakers to decide, they would say. To this we have only one answer. If they themselves were to fall sick and had to pay for their treatment, would they not want to think of the most cost effective therapy for themselves? If a Guideline does that for them, would they not thank medical practice and its practitioners that such care is indeed taken?

This is the crux of the issue. And no skirting it need be tolerated any longer.

## Universal Effectiveness And Variable Cost Effectiveness

Let us consider the third point.

Third, one could consider an assessment of effectiveness to be somewhat “universal.” In contrast, the determination of whether a therapy is acceptably cost-effective can certainly vary between jurisdictions (McFarlane, 2006).

Therapeutic effectiveness is ‘universal’ in a way, cost effectiveness and its acceptability is variable for different socio-economic and geographical areas. Agreed. Even then, let guideline layers lay down cost effectiveness in their geographical area of work. And match it with their judgement of universal effectiveness. Other workers at other places will study whether cost effectiveness as presented by the guidelines layers is equally applicable in their place. In barring a few cases, it is likely to be the same. How can the author say it can *certainly* vary? One has to carry out research to find out if it does. All one can say is it is *likely* to vary.

We agree there is an important element of collective judgement and discretion in deciding cost effectively. Let this judgement and discretion be exercised. Even if they err, they will have to present reasons why they decide what they do. Which others can analyse and correct. Why should that be unacceptable? The second, and important, fallout will be sponsors, and their henchmen, will not play a significant role any longer. This one scavenging effort will slough out the ulcer so it can hopefully heal.

## Three Groups: Most-Moderate-Least Cost Effective

Let, therefore, the three groups, Most Cost Effective, Moderately Cost Effective and Least Cost Effective be laid down. Different groups have a choice. Let's not forget amongst the most effective, they will be graded according to cost effectivity. Let's say both A and B are equally effective, but A is more cost effective than B. What then happens is that we offer an alternative to the patient. If cost is an important limiting consideration, he goes in for A. If cost is not, but exclusivity is, he goes in for B. Both have a choice to satiate their needs. It's like I go for a cup of tea in a roadside restaurant and pay Rs. 10. For a similar cup I pay Rs. 300 in a five star. Now, I have a choice to pay Rs. 10 or Rs. 300. Do not for a moment feel everyone will go for the Rs. 10 cup. There will always be those who seek the Rs 300 one. Well, let them have it their way. However, the seeker of a cup of tea will not be under any illusion that only the Rs. 300 cup of tea is real tea and will therefore have to pay through his nose to procure one. And all those who may be involved in marketing and projecting the Rs. 300 cup as the best tea will do so as the choice of connoisseurs or whatever, certainly not on the basis of evidence. What is happening today is evidence is being conveniently doctored to present only the Rs. 300 cup as tea and the others as, well, poor cousins.

This game must stop. For, it has very negative implications for patient welfare, patient confidence in biomedicine and future progress of biomedicine itself. Moreover, because of such games playing, the brighter minds that get into biomedical research have an alternative to progressing, apart from the straight and narrow path. And if such an alternative promises faster and easier returns, these brighter minds can gradually get persuaded to accept it as legitimate. This cannot but compromise the genuine progress of biomedical research. Also, patients’ confidence in therapy is likely to reduce and the inevitable suspiciousness of therapists’ intentions, resultant patients’ right activism and consequent legal tangles likely to increase. Alternatives to mainstream medicine are also likely to appear more attractive. All in all, chances are a number of serious road-blocks will hamper the forward march of this otherwise very useful and promising branch.

This is the danger we have to assiduously guard against.

## CPGs And Conflict Of Interest

Let us consider the final point:

Finally, an economics based approach would place guideline groups in a true conflict of interest between their patient advocacy role and their obligations to the health care payors (McFarlane, 2006).

This point needs elaboration. It means guideline groups will develop a conflict of interest if they consider cost effectiveness. Their basic loyalty is towards patient welfare and if they have to consider cost effectiveness, they may be forced to recommend less effective therapies just because they are less costly. And purely to satisfy people who pay for the health care.

While this is a distinct possibility, we wish to stress the exact opposite. When guideline groups cannot be kept on the straight and narrow path and till we find fool proof ways of keeping them thus, we have no option but to stress that under no circumstances they can mislead or get mislead themselves, in the name of patient advocacy, to recommend costly new therapies which have still not proved their effectiveness conclusively, nor get away with conflicted recommendations, which it is still not obligatory to reveal. A simple rider like making it mandatory for guideline groups to go into both effectiveness and cost effectiveness takes care that this is ensured. This is one conflict of interest which will be resolved only if its disclosure is openly promoted. For, in its name, hidden agendas can be conveniently forwarded unless numerous checks and balances are in place. Till we can get them working, a single simple emphasis on calculating cost effectivity will do the trick.

## Health Economics And Clinical Sections

Another objection needs to be considered as well:

It is important to recognize that the quality of the health economics section of a company's approval application could be lower than the clinical section, which could affect the subsequent conclusions about the drug (McFarlane, 2006).

While this may indeed be true, it need hardly detain us, because we are not concerned with the quality of the health economics section of a company, nor that of its clinical section. We are concerned with guideline groups *themselves* doing the effective and cost effective analyses. A company's application and its expertise has nothing to do with, or at least need have nothing to do with, what guideline groups conclude, unless of course both overlap. And woe betides biomedicine if they do.

The conclusion needs to be looked into:

The roles of guideline groups and drug review panels are both necessary and complimentary. Recognizing that the most effective therapies will not always be the most cost-effective leads to the appropriate expectation that guideline groups and drug review panels may reach opposite conclusions (McFarlane, 2006).

Indeed, it's true sometimes the most effective may not be the most cost effective. We would like to know why? Is it because the raw material and the processing is costly or is it because the drug/devise has to make the billions before it runs out of steam? This is a critical exercise, especially for the well-wishers of biomedical advance, who may be convinced by the above-mentioned argument. And in light of the conflicted interests involved, we think it is necessary that guideline groups also do cost analysis to salvage themselves. We think they need to reveal their conflicts of interest fully and desist from taking part if they are conflicted. They need to also resist any attempts to approve costly newer unproved therapies under the guise of effectivity. This they can do by considering and laying down most, moderate and least cost effective amongst the various prevalent therapies as of date.

Hence, it is necessary for guideline groups to find the most effective and also the most cost effective, of therapies and therapeutic clusters and to give reasons why they consider them such.

Then a guideline group really *guides*, which is its actual role.

## Small Improvement, Three-fold Price Hike

Below is the viewpoint of one who has worked with CDR and is a little more charitably disposed to CDRs than is Macfarlane (2006) we quoted above. Laupacis (2006) adds a different dimension to the whole debate. Talking specifically about insulin gargline being three times costlier than generic long-acting insulin, he says:

The reason CEDAC recommended against reimbursement was that the relatively small improvement in hypoglycemia was not felt to justify the drug's more than 3-fold price relative to NPH (neutral protamine Hagedorn) insulin (Laupacis, 2006).

There it is, in black and white. A small improvement true, but not warranting recommendation of a three fold costlier drug. Now, the CDR did it and we now know what to do. If the Guidelines members had realized it and brought it to the notice, how would it have harmed informed decision-making? Of course that it would possibly harm economic interests of sponsors and related agencies, including those sponsored, is a possible hidden agenda. The reason why cost is kept out of calculations now becomes explicit.

He goes on to say further, referring to minimization of bias in CPGs and Associations which sponsor it:

Clinical practice guidelines and reimbursement recommendations such as those of the Canadian Diabetes Association (CDA) and the Canadian Expert Drug Advisory Committee (CEDAC) about insulin glargine have a potentially great effect on clinical practice. Minimizing bias during their development is therefore at least as important as it is during clinical trials. A conflict-of-interest guideline is only one method of minimizing bias (Laupacis, 2006).

A conflict of interest revelation by guidelines members is only one method, true, but an important one and one which needs to be speedily implemented. And fully too and prospectively. Then it becomes that much more effective.

## Different Composition, Different Emphases

Talking of the different membership composition of CEDAC and CDA Guidelines, the author clearly has his finger on the pulse why they recommend differently and justify their respective positions:

CEDAC members are appointed by federal, provincial or territorial deputy ministers of health and are paid an honorarium by the CDR; public-drug-plan managers are allowed to observe CEDAC meetings; and CEDAC has no formal interaction with members of the public. In addition, CEDAC reports to the Board of the Canadian Coordinating Office of Health Technology Assessment (CCOHTA), which is made up entirely of representatives of the federal/provincial/ territorial Ministries of Health. No wonder a recent external assessment of the CDR found that members of advocacy groups representing people with various diseases called for greater public involvement in the CDR process. On the other hand, the CDA guidelines were sponsored by pharmaceutical and diagnostic companies, the methods of reviewing and summarizing the literature were not fully described and the potential conflicts of interest of authors are unknown (Laupacis, 2006).

CEDAC members, who talk of cost effectivity, are paid known amounts as honoraria. CDA Guidelines, which talk of effectivity, are sponsored by companies, their method of reviewing/summarizing literature are not clarified, their conflicts of interest not revealed and a drug three fold costlier is recommended. Does it take any great effort to read between the lines as to what this signifies?

This is the game we have to carefully expose and doggedly prevent from recurring.

Neither the guidelines nor the CDA letter of protest to the Canadian Ministers of Health about CEDAC's recommendation acknowledged the 3-fold price differential associated with insulin glargine compared with NPH insulin (Laupacis, 2006).

Isn't it obvious why it should be so? A statement such as this would weaken their case irreparably. And when you are part of an advocacy group, you advocate, you adopt a beneficial stand, you do not try to get to inconvenient truths.

No wonder those who pay for drugs are concerned that groups such as the CDA sometimes preferentially emphasize the evidence that supports their position and minimize the evidence that does not (Laupacis, 2006).

Emphasising evidence that supports and minimizing evidence that doesn't is advocacy at its best. And while it may suit the interests of those espoused, it is a moot point whether it serves the interests of biomedical advance and patient welfare, under which umbrellas all acts of omission and commission get carried out.

Interpreting the literature is not the same as summarizing it: interpretation inevitably incorporates an individual's values and perspectives. CEDAC's mandate is to make reimbursement recommendations from the perspective of the health care system, based not only upon a drug's effectiveness but also its cost-effectiveness (Laupacis, 2006).

Such should be the mandate of most, if not all, reimbursement systems. And paying for self-treatment systems as well. And we may add that till enough safe guards are in place with regard to CPGs, the one process that pre-empts much unfair means is calculation of cost-effectiveness by guideline layers themselves, which can be verified by bodies like CEDAC in particular and CDRs in general. What applies in this case in Canada is applicable in its essentials across other geographical areas.

I believe that the CDR process of reviewing and interpreting the available literature is as unbiased as possible (Laupacis, 2006).

While this is laudable, how much better would it be if we could say the same about Association CPG processes of reviewing and interpreting available literature? But to be able to say that, a thorough cleansing of the system and placing appropriate checks and balances in place, is obligatory.

Some effort in the direction of cost effectiveness evaluation is on. Eccles and Mason (2001) report on the cost-effectiveness sections of 11 guidelines. The study notes that, ‘*Unlike other areas of guideline development, there is little practical or theoretical experience to direct the incorporation of cost issues within clinical guidelines’* (p3). While noting that, ‘*Grading of recommendations of cost-effectiveness is in its infancy’*, (p62), it also notes that, along with effectiveness and quality of life data, *‘cost issues can successfully be represented as part of a broad profile of treatment attributes’* (p57).

## What Do We Do: AGREE And GAC?

There are both subjective and objective elements to a guideline. The objective are based on the quality of the evidence that support its recommendations, the subjective include the perspectives the authors bring to a guideline (Davis *et al*, 2006). The key to a good guideline is retaining the objectivity of the evidence and basing the subjectivity of perspectives on such objective evidence alone. When evidence gathering itself becomes subjective and the perspectives are based on such subjective evidence gathering, they no longer guide. They misguide and way lay, for extra-scientific forces guide them. A major part of our efforts today have to be directed to prevent such misguiding. Emphasis on cost considerations is one such important step to force the unscrupulous and open the eyes of the scrupulous (for they may unwitting subscribe to the actions of the former), as to what they need to do to remedy matters.

Another important initiative in this direction needs to be highlighted. The clinician today is bombarded with Guidelines. Which to choose? He cannot go through the burgeoning literature to judge for himself (though we think this should not become an excuse not to read critical literature in any field). So what does he do? Bodies like the AGREE Collaboration and the GAC come to his aid.

## AGREE And GAC

The AGREE Collaboration (AGREE stands for Appraisal of Guidelines, Research and Evaluation) has created and validated tools by which a clinician can himself rate guidelines by identifying factors that determine their quality. In using them, he considers factors like scope and purpose, objectives and patient population, whether involvement of all relevant stake-holders is ensured, its format and clarity and its applicability, wherein both organizational and cost barriers are considered. Most importantly, the clinician can also address bias issues by looking into the rigour and editorial independence of the guideline development process. Editorial independence is ensured by looking into whether the guidelines developers have maintained independence from funding agencies and other possible conflicts of interest. To ensure all this, besides the AGREE Instrument and its translation into ten languages, there are tools like Comparison of guidelines development programmes, Appraisal of individual recommendations, Content analysis of guidelines and also an AGREE Instrument Training Manual (AGREE, 2007).

However, AGREE itself requires some detailed work up by the individual clinician. Again a daunting task for most. For most clinicians neither have the aptitude, not the ability, to scale up the knowledge pyramid of burgeoning contemporary biomedical literature (scaling the knowledge pyramid, a phrase used by Davis et al, 2006). So what do we do? To facilitate this process occurs, a body like the GAC (Guidelines Advisory Committee) applies the AGREE criteria to individual guidelines and rates and endorses the best possible guideline (GAC, 2007).

Such an initiative needs to be duplicated at other places (this being an Ontario, Canada, initiative), in other countries, so guidelines remain less conflicted and become more relevant to local needs. What, however, must also be ensured is that CAG members and those of like bodies, do not themselves remain conflicted. An enlightened clinician-consumer, who keeps himself abreast of relevant literature from proper sources, is the greatest insurance against conflicted individuals taking biomedicine for a ride. By relevant literature we mean literature apart from pharma rep pamphlets and CMEs speeches. Proper sources include reading what those concerned with maintaining ethical standards in biomedicine write. As well as the write-ups of medical journalists.

Ethics is not a part time activity, like a prayer in the morning. Make token obeisance and carry on regardless. It is a full time barometer of all activities performed. Occasionally it appears a hindrance. And is irksome. In the long run, it is the best insurance against derailments and accidents that biomedicine seems so prone to nowadays due to profit considerations overriding all the rest.

## Concluding Remarks

1. A Conference on Guideline Standardization (COGS) was convened in April 2002 ‘to define a standard for guideline reporting that would promote guideline quality and facilitate implementation’. It includes items for standardization, conceptual issues, up gradation, conflict of interest, patient interest and systematization. Even items for individual preferences, choice or values are not neglected. Special mention must be made of items which specify disclosure of conflict of interest both in the Developer (including the organization that develops and the individuals involved in the guideline's formulation), as well as in the sponsor or funding source (and its role in developing and/or reporting the guideline).
2. Recommendations of CPGs and CDR panels are conflicting. One considers effectiveness, the other considers cost-effectiveness. However, CPGs do not adhere to established methodological standards, critical information that would attest to validity is regularly absent, explicit criteria to grade the scientific evidence that supports their recommendations is absent from 82% of guidelines, 87% are not in a position to report whether a systematic literature search was performed, 67% do not describe the type of professionals used in guidelines development, there is such marked variation in the quality of guidelines. Moreover, CPG guideline layers often are conflicted in their interests. The problem can be resolved to a large extent by taking a simple step: making CPG panelists go into cost effectiveness along with recommending Guidelines. What then happens is that they have to consider not only effectiveness but also costs. Now effectiveness can be fudged, cost cannot. Why? Because, what is the cost is well known. Therapies in Guidelines should be recommended and graded according to whether they are Most, Moderately or Least Cost Effective. For that CPGs will have to perform economic analysis as well. This will meet with resistance for obvious reasons.
3. When guideline groups cannot be kept on the straight and narrow path and till we find fool proof ways of keeping them thus, we have no option but to stress that under no circumstances they can mislead or get mislead themselves, in the name of patient advocacy, to recommend costly new therapies which have still not proved their effectiveness conclusively, nor get away with conflicted recommendations, which it is still not obligatory to reveal. A simple rider like making it mandatory for guideline groups to go into both effectiveness and cost effectiveness takes care that this is ensured.
4. The AGREE Collaboration (AGREE stands for Appraisal of Guidelines, Research and Evaluation) has created and validated tools by which clinicians can themselves rate guidelines by identifying factors that determine their quality. To facilitate this process, a body like the GAC (Guidelines Advisory Committee) applies the AGREE criteria to individual guidelines and rates and endorses the best possible guideline.

## Footnotes

* This was corrected to three times, as mentioned earlier. But that need not detract from the burden of the argument presented. -Eds.
